# A Systematic Review of Cost-Effectiveness Studies Reporting Cost-per-DALY Averted

**DOI:** 10.1371/journal.pone.0168512

**Published:** 2016-12-22

**Authors:** Peter J. Neumann, Teja Thorat, Yue Zhong, Jordan Anderson, Megan Farquhar, Mark Salem, Eileen Sandberg, Cayla J. Saret, Colby Wilkinson, Joshua T. Cohen

**Affiliations:** Center for the Evaluation of Value and Risk in Health, Institute for Clinical Research and Health Policy Studies, Tufts Medical Center, Boston, United States of America; Université Catholique de Louvain, BELGIUM

## Abstract

**Introduction:**

Calculating the cost per disability-adjusted life years (DALYs) averted associated with interventions is an increasing popular means of assessing the cost-effectiveness of strategies to improve population health. However, there has been no systematic attempt to characterize the literature and its evolution.

**Methods:**

We conducted a systematic review of cost-effectiveness studies reporting cost-per-DALY averted from 2000 through 2015. We developed the Global Health Cost-Effectiveness Analysis (GHCEA) Registry, a repository of English-language cost-per-DALY averted studies indexed in PubMed. To identify candidate studies, we searched PubMed for articles with titles or abstracts containing the phrases “disability-adjusted” or “DALY”. Two reviewers with training in health economics independently reviewed each article selected in our abstract review, gathering information using a standardized data collection form. We summarized descriptive characteristics on study methodology: e.g., intervention type, country of study, study funder, study perspective, along with methodological and reporting practices over two time periods: 2000–2009 and 2010–2015. We analyzed the types of costs included in analyses, the study quality on a scale from 1 (low) to 7 (high), and examined the correlation between diseases researched and the burden of disease in different world regions.

**Results:**

We identified 479 cost-per-DALY averted studies published from 2000 through 2015. Studies from Sub-Saharan Africa comprised the largest portion of published studies. The disease areas most commonly studied were communicable, maternal, neonatal, and nutritional disorders (67%), followed by non-communicable diseases (28%). A high proportion of studies evaluated primary prevention strategies (59%). Pharmaceutical interventions were commonly assessed (32%) followed by immunizations (28%). Adherence to good practices for conducting and reporting cost-effectiveness analysis varied considerably. Studies mainly included formal healthcare sector costs. A large number of the studies in Sub-Saharan Africa addressed high-burden conditions such as HIV/AIDS, tuberculosis, neglected tropical diseases and malaria, and diarrhea, lower respiratory infections, meningitis, and other common infectious diseases.

**Conclusion:**

The Global Health Cost-Effectiveness Analysis Registry reveals a growing and diverse field of cost-per-DALY averted studies. However, study methods and reporting practices have varied substantially.

## Introduction

Cost-effectiveness analysis (CEA), which quantifies the value of interventions in terms of costs per unit of health achieved, can help inform resource allocation decisions. Cost-per-quality-adjusted life year (QALY) gained (also referred to as cost-per-QALY) and cost-per-disability-adjusted life year (DALY) averted (also referred to as cost-per-DALY) studies are both approaches to cost-effectiveness analyses, in which the costs and effects of programs and at least one alternative are calculated and presented in a ratio of incremental cost to incremental effect [1]. QALYs and DALYs are metrics used to measure population health, which integrate morbidity and mortality, though there are some differences in how they are constructed [2]. Historically, QALYs have been applied predominantly in higher-income countries while DALYs have been used more extensively in global health analyses—e.g., the latter metric was favored by the WHO-CHOICE and Disease Control Priorities projects [3,4]. The World Bank and World Health Organization (WHO), along with academic researchers, developed the DALY in the 1990s as a generic measure of population health for assessing the burden imposed by a wide range of diseases and conditions [5,6]. DALYs capture both mortality in terms of years of life lost (YLL) and morbidity in terms of years of life with disability (YLD) [7]. The YLD component represents a duration that is scaled by disability severity weights that range from 0 (no adverse impact on quality of life) to 1 (burden equivalent in preference to being dead) [8,9]. The Global Burden of Disease project has developed standardized weights to facilitate comparisons across regions and countries [10]. The number of cost-per-DALY studies has grown in recent years, with many of these analyses focusing on health interventions in low- and middle-income countries. However, there has been no systematic attempt to characterize the literature and its evolution.

This article describes a systematic review of the cost-per-DALY literature as well as the creation of a new database that catalogs these studies, detailing the interventions, conditions, and target populations investigated, and the quality and consistency of the methods employed. The goals are to help policy makers and researchers understand the best opportunities to improve population health in their jurisdictions given resource constraints and to help standardize methods in the field, thus improving quality and comparability across cost-per-DALY studies. We also examine trends for the types of interventions, methods and reporting practices. Finally, we investigate the extent to which cost-per-DALY studies have targeted diseases imposing the largest population burden.

## Methods

### Development of the GHCEA registry

The Center for the Evaluation of Value and Risk in Health (CEVR) at Tufts Medical Center developed the Global Health Cost-Effectiveness Analysis (GHCEA) Registry, a repository of English-language cost-per-DALY studies indexed in PubMed. The Registry, supported by a grant from the Bill & Melinda Gates Foundation, is a free, online resource (http://healtheconomics.tuftsmedicalcenter.org/ghcearegistry/) for researchers, policy makers, and other parties. The current version of the website allows searches of various aspects of published cost-per-DALY studies, including titles of papers and cost-per-DALY ratios, stratified by characteristics such as disease type, region, country and subpopulation (e.g., children). Future versions of the website will provide further enhancements to expand the scope and improve usability.

### Systematic search of the literature

**Fig 1:**
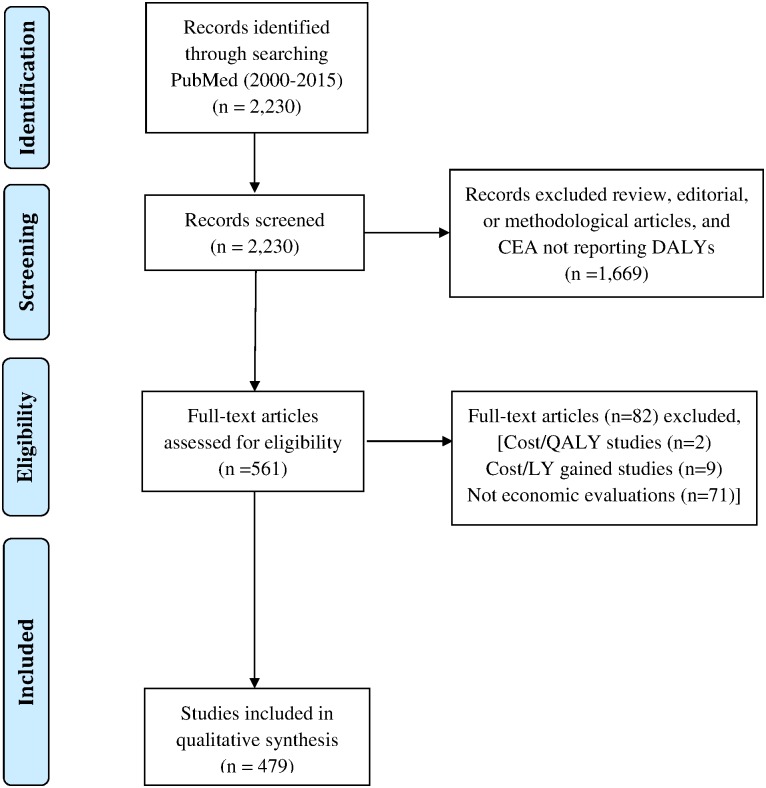
Details of search and data collection strategy.

The GHCEA Registry includes published English-language studies that report original cost-per-DALY assessments from 2000–2015 (regular updates are planned for the future). It builds on research protocols developed for the Tufts Medical Center CEA Registry, a database of published cost-per-QALY analyses (QALYs) [11,12]. To identify candidate studies, we systematically searched PubMed for articles with titles or abstracts containing the phrases “disability-adjusted” or “DALY”. We then screened candidate abstracts to determine if the referenced studies contain an original cost-per-DALY estimate. We excluded review, editorial, or methodological articles, and CEAs that do not quantify health impacts in terms of DALYs. Fig 1 details the search and data collection strategy.

Two reviewers with training in health economics independently reviewed each article selected in our abstract review, gathering information using a standardized data collection form. Reviewers extracted data relating to study design and methodology, interventions, and outcomes. The two reviewers then convened for a consensus audit to resolve potential discrepancies and to ensure the quality of the collected information.

For each article, we described the health intervention evaluated, the intervention to which it was compared (the “comparator”), and the target population. We also collected information for each study on: 1) intervention type (e.g., pharmaceutical, immunization, surgery); 2) country of focus; 3) funding source; 4) accuracy of the reported cost-per-DALY; 5) time horizon; 6) perspective (e.g., societal, health care sector or health care payer); 7) types of costs included (e.g., formal health care sector, informal health care sector, non-health care sector); 8) discount rate, if any; 9) age-weighting; 10) currency and currency year; 11) inclusion or exclusion of additional costs associated with greater longevity conferred by the treatment; 12) type of sensitivity analysis performed, and 13) the specified cost-per-DALY threshold value reported as a benchmark to assess cost-effectiveness favorability (ratios with values below the threshold indicate accrual of health benefits at a relatively low cost-per-DALY, hence suggesting the underlying intervention has “good value”). For each article, we also provided an overall quality score on a scale from 1 (low) to 7 (high), based on factors, such as whether articles present a correct computation of the ICERs, a complete characterization of uncertainty, and appropriate reporting of the intervention, comparator and target population.

**Fig 2:**
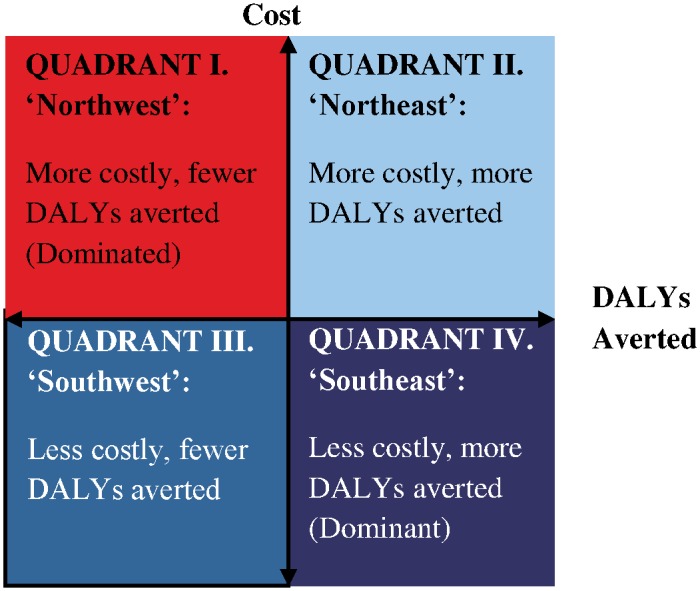
Cost-effectiveness ratio quadrant.

We recorded the cost-effectiveness ratio value, and when available, incremental costs (the ratio’s numerator) and health effects (DALYs—the ratio’s denominator). We also identified the quadrant in the “cost-effectiveness plane” containing each ratio. As illustrated in Fig 2, the northeast and northwest quadrants contain ratios for interventions that increase costs. The northeast and southeast quadrants contain ratios for interventions that improve health (avert DALYs). So, the northeast quadrant contains the intersection of these two groups (ratios for interventions that increase costs but also improve health). The southeast quadrant contains ratios for interventions that both improve health and reduce costs. The northwest quadrant contains ratios for interventions that increase costs and make health worse.

To facilitate comparisons of ratios across countries, we converted costs to 2014 US dollars using Purchasing Power Parity (PPP) reported by the World Bank [13] and adjusted for inflation using the “All Urban Consumers” component of the US Bureau of Labor Statistics Consumer Price Index [14].

Finally, we characterized uncertainty information reported for each ratio. This information might include a cumulative distribution of plausible values (sometimes referred to as a cost-effectiveness acceptability curve), a ratio confidence interval, or sensitivity analysis results (the range of cost-effectiveness ratio values associated with alternative assumptions).

We recorded each health state and the disability weight value associated with it, as reported in the study. We also recorded the data source (primary or secondary) for the reported disability weight.

## Analyses

We explored trends over time for various study characteristics for two time periods: 2000 to 2009 and 2010 to 2015. For disease, we used the International Statistical Classification of Diseases and Related Health Problems, tenth revision (ICD-10) and the Global Burden of Disease (GBD) initiative classification system [15,16]. For intervention location, we used the GBD classification of countries by region and super region [3]. These regions group countries by geography and by population characteristics (in particular, country income level). We described study and methodological characteristics, including presentation of cost-effectiveness acceptability curves, correct incremental cost-effectiveness calculation, discounting, presentation of sensitivity analyses, and proper identification of study perspective. Finally, we analyzed the association between the top 10 diseases by burden and the number of 2000–2015 cost-per-DALY studies targeting each disease area. We conducted this analysis for the entire literature and looked at associations within the three most studied regions (Sub-Saharan Africa; High-Income Countries; and Southeast Asia, East Asia, and Oceania).

## Results

### Growth and characteristics of cost-per-DALY studies, 2000–2015

**Fig 3:**
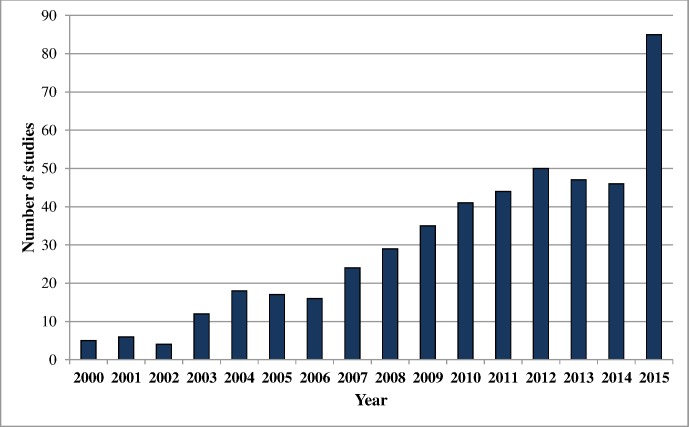
Growth in published cost-per-DALY studies, 2000–2015.

We identified 479 cost-per-DALY studies published from 2000 through 2015. The number of cost-per-DALY studies published each year increased markedly after 2007 (Fig 3). Notably, 85 studies were published in 2015, compared with 46 in 2014. The spike in 2015 appears to be an unusual increase in the number of new journals publishing cost-per-DALY studies (21 new journals in 2015 compared to 10 new journals in 2014) and in an increase in the number of studies at certain journals—e.g., *Vaccine* published 15 cost-per-DALY studies in 2015, up from 3 studies in 2014. Studies from Sub-Saharan Africa comprised the largest portion of published studies (34%), followed by studies in high-income countries (21%) and countries in Southeast Asia, East Asia, and Oceania (13%) (Table 1). The most common GBD disease classifications were communicable, maternal, neonatal, and nutritional disorders (67%), followed by non-communicable diseases (28%) and injuries (2%). The most common ICD-10 classifications were infectious and parasitic diseases (56%), followed by endocrine, nutritional and metabolic diseases (8%), and mental and behavioral disorders (8%). The most commonly assessed interventions included pharmaceuticals (32%), immunizations (28%), and health education and behavior programs (22%).

**Table 1:** Characteristics of published cost-per-DALY studies, 2000–2015 (n = 479).

	2000–2009	2010–2015	Overall
Number of studies	n = 166	n = 313	n = 479
Super Regions*			
Sub-Saharan Africa	27%	37%	34%
High income	24%	20%	21%
Southeast Asia, East Asia, and Oceania	14%	12%	13%
Latin America and Caribbean	8%	11%	10%
South Asia	8%	10%	9%
North Africa and Middle East	1%	3%	2%
Central Europe, Eastern Europe, and Central Asia	2%	2%	2%
Multiple regions^#^	20%	10%	13%
**Disease (ICD-10)**			
Infectious and parasitic diseases	58%	55%	56%
Endocrine, nutritional and metabolic diseases	8%	8%	8%
Mental and behavioral disorders	11%	7%	8%
Circulatory system diseases	4%	5%	5%
Pregnancy, childbirth and the puerperium	2%	6%	5%
Neoplasms	5%	2%	3%
Other	11%	16%	15%
**Diseases- GBD Classification**			
***Communicable*, *maternal*, *neonatal*, *and nutritional disorders***	67%	66%	67%
Diarrhea, lower respiratory infections, meningitis, and other common infectious diseases	22%	21%	21%
HIV/AIDS and tuberculosis	19%	20%	20%
Neglected tropical diseases and malaria	11%	10%	10%
Nutritional deficiencies	6%	4%	4%
Maternal disorders	2%	4%	4%
Neonatal disorders	1%	3%	2%
Other communicable, maternal, neonatal, and nutritional disorders	8%	8%	8%
***Non-communicable diseases***	27%	28%	28%
Mental and behavioral disorders	11%	7%	8%
Neoplasms	6%	5%	6%
Diabetes, urogenital, blood, and endocrine diseases	3%	6%	5%
Cardiovascular and circulatory diseases	4%	5%	5%
Other non-communicable diseases	4%	7%	6%
***Injuries***	1%	3%	2%
Transport injuries	1%	2%	1%
Other	1%	1%	1%
Other**	4%	4%	4%
Intervention*			
Pharmaceutical	36%	29%	32%
Immunization	30%	27%	28%
Health education and behavior	26%	20%	22%
Care delivery	15%	15%	15%
Screening	12%	15%	14%
Maternal/neonatal care	10%	10%	10%
Other	49%	51%	51%
Study funder*			
Government	49%	46%	47%
Foundation	31%	36%	34%
Academic institutions	8%	15%	13%
Healthcare organizations^	16%	7%	10%
Pharmaceutical industry	4%	4%	4%
None	2%	8%	6%
Not Determined	22%	14%	17%
Other	8%	10%	9%
Prevention stage*			
Primary	61%	58%	59%
Secondary	17%	18%	18%
Tertiary	42%	36%	38%

* Not mutually exclusive

Super regions and GBD classification for diseases are as reported in the Global Burden of Disease (GBD) study, 2010 [15].

^#^ Multiple regions indicate that an estimate in a single study was provided for multiple countries across different regions.

^ Health care organizations includes insurance companies, hospitals, Health Maintenance Organizations (HMOs), WHO.

** Some interventions (e.g., telemedicine, surgical improvements) were not specific to a particular disease, but pertained to multiple diseases.

More than half of the studies (59%) evaluated primary prevention interventions (e.g. disease screening), followed by tertiary prevention interventions (treatments) (38%) and secondary prevention interventions (18%) (interventions targeting high-risk individuals who have not yet developed clinical disease). Government (47%) and foundations (34%) were the most common sources of funding. Pharmaceutical industry sponsors funded 4% of published studies.

### Top journals publishing cost-per-DALY studies

The top journals publishing cost-per-DALY studies include *Vaccine* (47 studies), *PLOS One* (46), and WHO’s *Bulletin of the World Health Organization* (18) (Table 2). In total, 151 journals published at least one cost-per-DALY study from 2000–2015.

**Table 2:** Top journals publishing cost-per-DALY studies, 2000–2015.

Journal Name	Number of articles
*Vaccine*	47
*PLOS ONE*	46
*Bulletin of the World Health Organization*	18
*World Journal of Surgery*	17
*Cost Effectiveness and Resource Allocation*	14
*Health Policy and Planning*	14
*BMJ*	12
*PLOS Medicine*	12
*The Journal of Infectious Diseases*	10
*BMC Public Health*	9
*Value in Health*	9
*Australian & New Zealand Journal of Psychiatry*	8
*International Journal of Tuberculosis and Lung Disease*	8
*The Lancet Global Health*	8
*Tropical Medicine & International Health*	8

### Methodological and reporting practices

Approximately 30% of studies presented results from a societal perspective (studies including costs related to formal healthcare and at least one category of informal healthcare costs, i.e., patient or informal caregiver time; transportation, productivity loss, income loss, or costs in non-healthcare sectors) (Table 3). Most studies mentioned a WHO-recommended cost-effectiveness benchmark threshold of 1 times gross domestic product (GDP) per capita and 3 times GDP per capita (30%). Studies most often used a discount rate of 3 percent for costs (65%) and DALYs (73%). Most studies reported sensitivity analyses (92%).

**Table 3:** Methodological and reporting practices of published cost-per-DALY studies, 2000–2015.

Variables	2000–2009 n = 166	2010–2015 n = 313	Total n = 479
**Study perspective**^#^			
Societal	29%	31%	30%
Health care sector	10%	9%	9%
Health care payer	60%	58%	58%
Not stated/Other	1%	2%	2%
Reviewer and author agree on study perspective**			
Yes	58%	73%	68%
**Cost-effectiveness threshold mentioned**			
GDP per capita	32%	25%	24%
3X GDP per capita	4%	6%	5%
Both GDP and 3X GDP per capita	28%	36%	30%
None	33%	17%	23%
Other	31%	22%	26%
**Acceptability curve presented**			
Yes	8%	20%	16%
**Disaggregate costs and DALYs reported**			
Yes	96%	96%	96%
**Disaggregate costs and DALYs compute reported ICER**			
Yes	78%	74%	75%
Cost discount rate*			
None	4%	4%	4%
3% annual rate	62%	66%	65%
Other	9%	5%	6%
Not applicable^	7%	7%	7%
Could not be determined	18%	17%	17%
DALY discount rate*			
None	2%	4%	3%
3% annual rate	73%	74%	73%
Other	8%	5%	6%
Not applicable^	5%	4%	4%
Could not be determined	12%	14%	13%
Age-weighting used*			
Yes	36%	24%	28%
**Sensitivity analysis presented**			
Yes	90%	92%	92%
Univariate^+^	75%	82%	79%
Multivariate^+^	62%	59%	60%
Probabilistic^+^	47%	49%	48%
**Disclosed funding**			
Yes	78%	86%	83%
**Mean quality score**^++^	4.75	5.00	4.89

^+^ Categories are not mutually exclusive

^#^ Societal perspective includes studies that incorporated formal healthcare costs (medications, hospitalizations) + at least one informal healthcare costs (productivity, caregiver time, income loss) or non-healthcare costs (other sectors- education, legal).

Health care sector perspective includes costs related to medical treatment (formal healthcare) + out-of-pocket costs incurred by patients.

Health care payer perspective includes costs incurred by a (typically 3rd party) health care payer.

The perspective noted here is that judged by our reviewers not by study authors (see note to this table below).

** Our reviewers of the cost-per-DALY studies sometimes disagreed with the study authors on the actual perspective taken in the analysis. For example, study authors may have claimed that they took a “societal perspective” when in fact they only accounted for costs in the health sector, thus following a “health care sector” perspective.

* Global Burden of Disease (GBD) recommendations are as follows- GBD 1990- age-weighting, discount rate = 3; GBD 2001-2- no age-weighting, discount rate = 3; GBD 2004- age-weighting, discount rate = 3; GBD 2010- no age-weighting, discount rate = 3

^ Discount rates for DALYs or costs are considered not applicable if the time horizon reported in the study is less than or equal to 1 year.

^++^ Overall score for study quality ranges from 1 (low) to 7 (high).

Adherence to good practices for conducting and reporting cost-effectiveness analysis improved for several items between the 2000s and the period 2010–15 (Table 3). For example, appropriate reporting of study perspective increased from 58% during the period 2000–2009 to 73% from 2010–2015; for these periods, presentation of acceptability curves increased from 8% to 20%; disclosure of funding increased from 78% to 86%; identification of a cost-effectiveness benchmark threshold increased from 67% to 83%. With the change in GBD methods away from age-weighting, the proportion of studies using age-weighting declined from 36% during the period 2000–2009 to 24% during the period 2010–2015. The quality score was roughly unchanged over the time period (4.8 for 2000–2009 studies versus 5.0 for the 2010–2015 studies).

### Types of costs included in cost-per-DALY studies

Although virtually all studies (97%) incorporated healthcare sector costs (Table 4), inclusion of other types of costs varied considerably. Only 33% of studies included informal health care sector costs, such as transportation-related costs (22%), informal caregiver time (12%), or patient time costs (11%). A small proportion (5%) of studies included costs from sectors other than health care (e.g. education (2%), environment (1%), and legal (0.4%)). Many studies (63%) included intervention implementation costs, such as the personnel salaries (47%), infrastructure costs (43%), and administrative costs (43%).

**Table 4:** Types of costs included in published cost-per-DALY studies, 2000–2015.

Costs Included^+^	2000–2009 (n = 166)	2010–2015 (n = 313)	Overall (n = 479)
**Formal healthcare sector**	99%	97%	97%
Direct medical	99%	96%	97%
Out-of-pocket	17%	26%	23%
**Informal healthcare sector**	29%	36%	33%
Transportation	20%	23%	22%
Informal caregiver time	8%	13%	12%
Patient time	8%	12%	11%
Income loss	8%	10%	9%
Productivity	5%	10%	9%
Other***	10%	9%	9%
Non-healthcare sector*	5%	5%	5%
Education	2%	2%	2%
Environment	1%	1%	1%
Legal	0%	1%	0.4%
Housing	1%	0%	0.4%
Other	4%	4%	4%
Implementation costs**	69%	60%	63%
Personnel salary	54%	43%	47%
Infrastructure	51%	38%	43%
Administrative	48%	41%	43%
Other****	29%	21%	24%

^+^ Categories are not mutually exclusive

* Non-healthcare sector includes costs related to sectors outside of healthcare (e.g., judicial, housing, education, legal)

*** Other includes costs associated with patient’s accommodation and food

** Implementation costs includes the following- Personnel salaries- includes salaries associated with staff involved in the intervention; Infrastructure- costs associated with setup of the intervention; Administrative costs- Expenses incurred in controlling, directing and managing the intervention; Other- Other costs related to the implementation of the intervention. E.g. training, surveillance, advertising

**** Other includes costs associated with personnel transportation, surveillance, and marketing

### Association of cost-per-DALY studies with burden of disease across geographic regions

**Fig 4:**
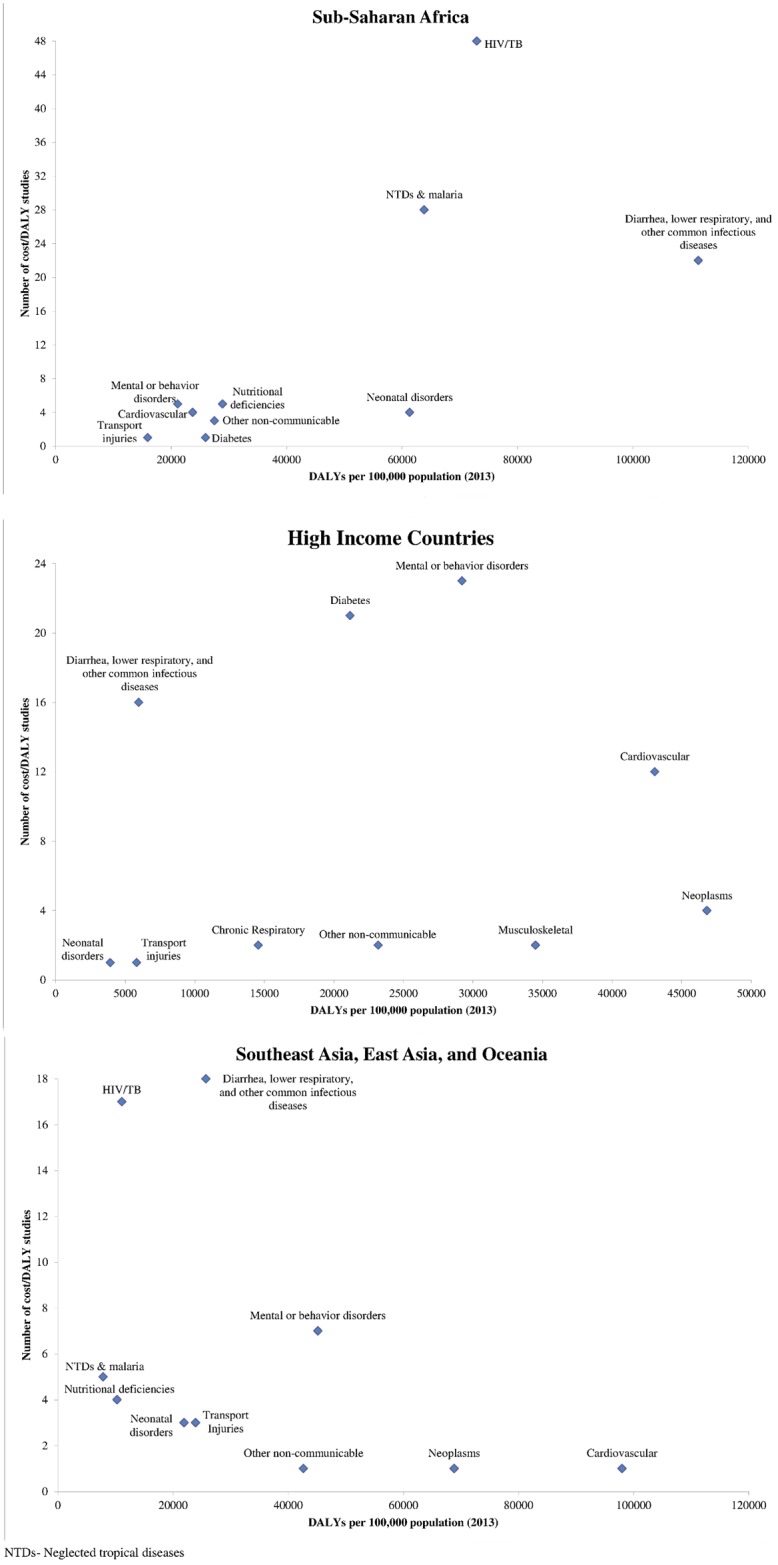
Burden of disease (2013) vs. number of cost-per-DALY studies (2000–2015) by Global Burden of Disease (GBD) super regions [3], for most common diseases from 2013. Legend- Sub-Saharan Africa includes all African countries with the exception of North Africa. High-income countries include those in Southern Latin America, Western Europe, high-income North America, Australia, and High Income Asia Pacific.

Fig 4 illustrates the extent to which the cost-per-DALY literature has addressed the largest health problems in various GBD super regions. A moderately large number of Sub-Saharan Africa studies address high burden infectious conditions such as HIV/AIDS, tuberculosis, neglected tropical diseases and malaria, and diarrhea, lower respiratory infections, meningitis, and other common infectious diseases. Few studies address other high burden disease areas, such as nutritional deficiencies and neonatal disorders. Few high-income country cost-per-DALY studies address certain high burden conditions in that region, including neoplasms and musculoskeletal conditions. Few cost-per-DALY studies address Southeast Asia, East Asia, and Oceania. Few of those that have been conducted address high burden conditions, such as cardiovascular disease, cancer, diabetes and chronic respiratory infections. On the other hand, relative to disease burden, a relatively large number of studies have focused on HIV/AIDS.

## Discussion

The Global Health Cost-Effectiveness Analysis Registry catalogs 479 cost-per-DALY studies published from 2000–2015 and reveals a growing and diverse field. Investigators have used the cost-per-DALY framework to examine a wide range of interventions, diseases, and settings, with a heavy focus on interventions targeting infectious diseases.

Though there has been some improvement in adherence to good practices for conducting and reporting cost-effectiveness analysis, study methods have varied substantially. Notably, studies include different types of costs. Because these differences suggest the need for greater standardization, efforts like the Gates Reference Case and the report of the Second U.S. Panel on Cost-Effectiveness in Health and Medicine are welcome [2,17].

Most of the studies have focused on Sub-Saharan Africa and Asia and in general, have focused on infectious and parasitic diseases. This focus may reflect the priorities of funding agencies and disease burden, though our study also highlights certain diseases and conditions (e.g., neonatal disorders, cardiovascular disease, injuries) that are under-represented in the cost-effectiveness literature relative to the burden they impose. These disparities underscore the need for governments, foundations, and researchers to reexamine health economic research priorities.

These findings on the growth and variation in the cost-per-DALY literature are similar in many ways to reviews of the cost-per-QALY literature. Research has shown rapid growth in the number of published cost-per-QALY studies, with applications to diverse diseases and interventions, but also substantial variability in reporting practices as well disparities between certain conditions (e.g., injuries) with high burden but which have had few associated cost-effectiveness analyses [18].

As our study shows, cost-per-DALY studies focus mostly on countries in Africa, and Asia. In contrast, cost-per-QALY studies focus primarily on the United States and Europe [18]. The differences seem to be a historical “accident,” rather than reflecting a strong rationale or intention. QALYs were developed and nurtured in the US and Europe. On the other hand, DALYs were developed to measure global burden of disease and were popularized in the global health community. In future work, we will examine how the use of cost-per-DALY estimates and cost-per-QALY estimates might differentially influence prioritization of interventions. We will also investigate the extent to which estimates developed for one country or region can be applied generalized to other settings. Finally, we will consider expanding our work to include other databases beyond PubMed, and to include articles published in languages other than English.

## Supporting Information

S1 Checklist PRISMA checklist. (DOC)

## References

[1] Sanders GD, Neumann PJ, Basu A, Brock DW, Feeny D, Krahn M et al Recommendations for Conduct, Methodological Practices, and Reporting of Cost-effectiveness Analyses: Second Panel on Cost-Effectiveness in Health and Medicine. JAMA 2016; 316(10):1093–1103. 10.1001/jama.2016.12195 27623463

[2] Neumann PJ, Sanders GD, Russell LB, Siegel JE, Ganiats TG. Cost-Effectiveness in Health and Medicine. 2nd Edition ed. New York, NY: Oxford University Press; 2016.

[3] Murray CJ, Ezzati M, Flaxman AD, Lim S, Lozano R, Michaud C et al GBD 2010: design, definitions, and metrics. Lancet 2012; 380(9859):2063–2066. 10.1016/S0140-6736(12)61899-6 23245602

[4] Global, regional, and national age-sex specific all-cause and cause-specific mortality for 240 causes of death, 1990–2013: a systematic analysis for the Global Burden of Disease Study 2013. Lancet 2015; 385(9963):117–171. 10.1016/S0140-6736(14)61682-2 25530442 PMC4340604

[5] Murray CJ, Salomon JA, Mathers CD, Lopez AD. Summary measures of population health: concepts, ethics, measurement and applications. Geneva: 2002.

[6] World Bank. World Development Report 1993: Investing in Health. Oxford University Press, editor 1993. [13 June 2016]. https://openknowledge.worldbank.org/handle/10986/5976

[7] Devleesschauwer B, Havelaar AH, Maertens de NC, Haagsma JA, Praet N, Dorny P et al DALY calculation in practice: a stepwise approach. Int J Public Health 2014; 59(3):571–574. 10.1007/s00038-014-0553-y 24748107

[8] World Health Organization. Disability weights, discounting and age weighting of DALYs. [13 June 2016]. http://www.who.int/healthinfo/global_burden_disease/daly_disability_weight/en/

[9] World Health Organization. Metrics: Disability-Adjusted Life Year (DALY). [13 June 2016]. http://www.who.int/healthinfo/global_burden_disease/metrics_daly/en/

[10] Salomon JA, Haagsma JA, Davis A, de Noordhout CM, Polinder S, Havelaar AH et al Disability weights for the Global Burden of Disease 2013 study. Lancet Glob Health 2015; 3(11):e712–e723. 10.1016/S2214-109X(15)00069-8 26475018

[11] Center for Evaluation of Value and Risk in Health TMC. Tufts Medical Center Cost Effectiveness Analysis Registry. www.cearegistry.org. [13 June 2016]. www.cearegistry.org

[12] Thorat T, Cangelosi M, Neumann PJ. Skills of the trade: the Tufts Cost-Effectiveness Analysis(CEA) Registry. J Benefit-Cost Analysis 2012; 3:1–9.

[13] World Bank. PPP Conversion Factor, GDP (LCU per international dollar). [13 June 2016]. http://data.worldbank.org/indicator/PA.NUS.PPP

[14] U.S. Department Of Labor—Bureau of Labor Statistics. Consumer Price Index: All Urban Consumers (CPI-U). [13 June 2016]. http://www.bls.gov/cpi/cpid1512.pdf

[15] Murray CJ, Vos T, Lozano R, Naghavi M, Flaxman AD, Michaud C et al Disability-adjusted life years (DALYs) for 291 diseases and injuries in 21 regions, 1990–2010: a systematic analysis for the Global Burden of Disease Study 2010. Lancet 2012; 380(9859):2197–2223. 10.1016/S0140-6736(12)61689-4 23245608

[16] World Health Organization. International Classification of Diseases (ICD). [13 June 2016]. http://www.who.int/classifications/icd/en/

[17] Bill & Melinda Gates Foundation, NICE International. Gates Reference Case 2014. [13 June 2016]. https://www.nice.org.uk/Media/Default/About/what-we-do/NICE-International/projects/Gates-Reference-case-what-it-is-how-to-use-it.pdf

[18] Neumann PJ, Thorat T, Shi J, Saret CJ, Cohen JT. The changing face of the cost-utility literature, 1990–2012. Value Health 2015; 18(2):271–277. 10.1016/j.jval.2014.12.002 25773562

